# Evaluation of the Prognostic Value of Quantitative and Visual Fluorodeoxyglucose Positron Emission Tomography/Computed Tomography (FDG PET/CT) Parameters in Superficial Esophageal Squamous Cell Carcinoma

**DOI:** 10.7759/cureus.96941

**Published:** 2025-11-16

**Authors:** Arman Nessipkhan, Reiko Ideguchi, Yuko Akazawa, Hisaya Tanaka, Darkhan Uzbekov, Takashi Kudo

**Affiliations:** 1 Department of Radioisotope Medicine, Atomic Bomb Disease Institute, Nagasaki University, Nagasaki, JPN; 2 Department of Histology and Cell Biology, Nagasaki University Graduate School of Biomedical Sciences, Nagasaki University, Nagasaki, JPN; 3 Department of Surgery, Fukuda Yutaka Surgery Clinic, Nagasaki, JPN; 4 Department of Histology and Cytology, Astana Medical University, Astana, KAZ

**Keywords:** esophageal squamous cell carcinoma, fdg pet-ct, prognosis, quantitative, suvmax

## Abstract

Introduction: Early diagnosis is crucial for improving outcomes in esophageal cancer, as prognosis is strongly dependent on the stage at detection. This study evaluated the prognostic value of quantitative and visual 18F-fluorodeoxyglucose positron emission tomography/computed tomography (FDG PET/CT) parameters in patients with superficial esophageal squamous cell carcinoma (SESCC).

Materials and methods: A retrospective cohort of 95 patients with clinically confirmed SESCC who underwent pre-treatment FDG PET/CT was analyzed. Patients were stratified into FDG-positive and FDG-negative groups using both visual assessment and quantitative thresholds. Quantitative parameters included maximum standardized uptake value (SUVmax), metabolic tumor volume (MTV), and total lesion glycolysis (TLG). Associations with overall survival and pathological TNM classification were assessed.

Results: Quantitative analysis using SUVmax (threshold = 3.0) demonstrated significant prognostic stratification, with FDG-negative patients exhibiting superior overall survival compared to FDG-positive patients (p = 0.0264, log-rank test). Visual assessment did not yield significant survival discrimination (p = 0.3222). No significant correlations were identified between FDG-derived parameters and pathological or clinical TNM stages.

Conclusion: Quantitative FDG PET/CT parameters, particularly SUVmax, provide superior prognostic information compared to visual assessment in SESCC. Incorporating quantitative metabolic imaging into the initial evaluation of early-stage esophageal cancer may enhance risk stratification and inform treatment planning.

## Introduction

Esophageal cancer remains one of the leading causes of cancer-related mortality worldwide, largely due to its poor prognosis [1]. Early detection is critical for improving patient outcomes; however, diagnosing esophageal cancer at an early stage is challenging, as visual findings alone are often insufficient [2]. In potentially curable cases, timely diagnosis is essential to enable surgical or endoscopic resection, thereby reducing morbidity and mortality [3].

Endoscopy is currently the gold standard for diagnosing superficial esophageal cancer, yet it has notable limitations in predicting prognosis. 18F-fluorodeoxyglucose positron emission tomography/computed tomography (FDG PET/CT) is well established for staging advanced esophageal cancer, but its value in early superficial disease is less clear. Due to the relatively low spatial resolution of PET, its utility for detecting or risk-stratifying superficial lesions has been questioned. Indeed, Little et al. reported no prognostic benefit of FDG PET in superficial esophageal cancer [4].

Superficial esophageal squamous cell carcinoma (SESCC) poses a particular diagnostic and prognostic challenge. While FDG PET/CT has been used primarily for qualitative (visual) interpretation, this approach is limited by interobserver variability and insufficient sensitivity at early stages. Emerging evidence suggests that quantitative metabolic parameters, such as maximum standardized uptake value (SUVmax), metabolic tumor volume (MTV), and total lesion glycolysis (TLG), may provide more objective and reproducible prognostic insights.

Therefore, the aim of this study was to compare the prognostic value of visual and quantitative FDG PET/CT parameters, particularly SUVmax, in patients with SESCC. We hypothesize that quantitative measures offer superior prognostic discrimination and may establish a more reliable basis for risk stratification and clinical decision-making compared with visual assessment alone.

## Materials and methods

Ethics

This study was conducted in accordance with the ethical standards of the institutional and national research committees and with the principles of the 1964 Declaration of Helsinki and its later amendments. The study protocol was approved by the Ethics Committee of Nagasaki University Hospital (Protocol No. 21021511). Owing to the retrospective and non-invasive nature of the analysis, and because all data were anonymized, the requirement for written informed consent was waived.

Patients

We retrospectively reviewed 95 consecutive patients with endoscopically confirmed superficial esophageal cancer who underwent FDG PET/CT prior to treatment at Nagasaki University Hospital between July 2010 and June 2021. To maintain a homogeneous study population, patients with esophageal adenocarcinoma (n=14) and those with large tumor burden inconsistent with superficial disease (n=7) were excluded. Examinations with severe motion or technical artifacts and cases with incomplete PET/CT or essential clinical data were not included in the final analysis. This approach ensured that only studies of sufficient image quality and complete clinical information were evaluated. The final cohort included patients with SESCC. Clinical and pathological staging was performed according to the eighth edition of the TNM Classification of Malignant Tumors [5]. Demographic data, treatment records, survival status, and histopathological information were extracted from medical records.

PET/CT protocol

All patients underwent 18F-FDG PET/CT using a Siemens mCT scanner (Siemens Healthcare, Germany). Patients fasted for at least five hours prior to tracer administration. A standard dose of approximately 200 MBq of FDG was administered intravenously, and image acquisition commenced 60 minutes post-injection. Scanning was performed from the mid-thigh to the skull base. Image reconstruction used the ordered subset expectation maximization (OSEM) algorithm with two iterations and 24 subsets, a 200 × 200 matrix, a field of view of 815 mm, and a 6-mm Gaussian post-reconstruction filter. Standardized uptake values (SUVs) were normalized to body weight. All PET/CT examinations were acquired on the same Siemens mCT scanner, which underwent routine daily quality control and periodic calibration in accordance with institutional and manufacturer protocols to maintain consistency of SUV measurements over time.

Image analysis

**Figure 1:**
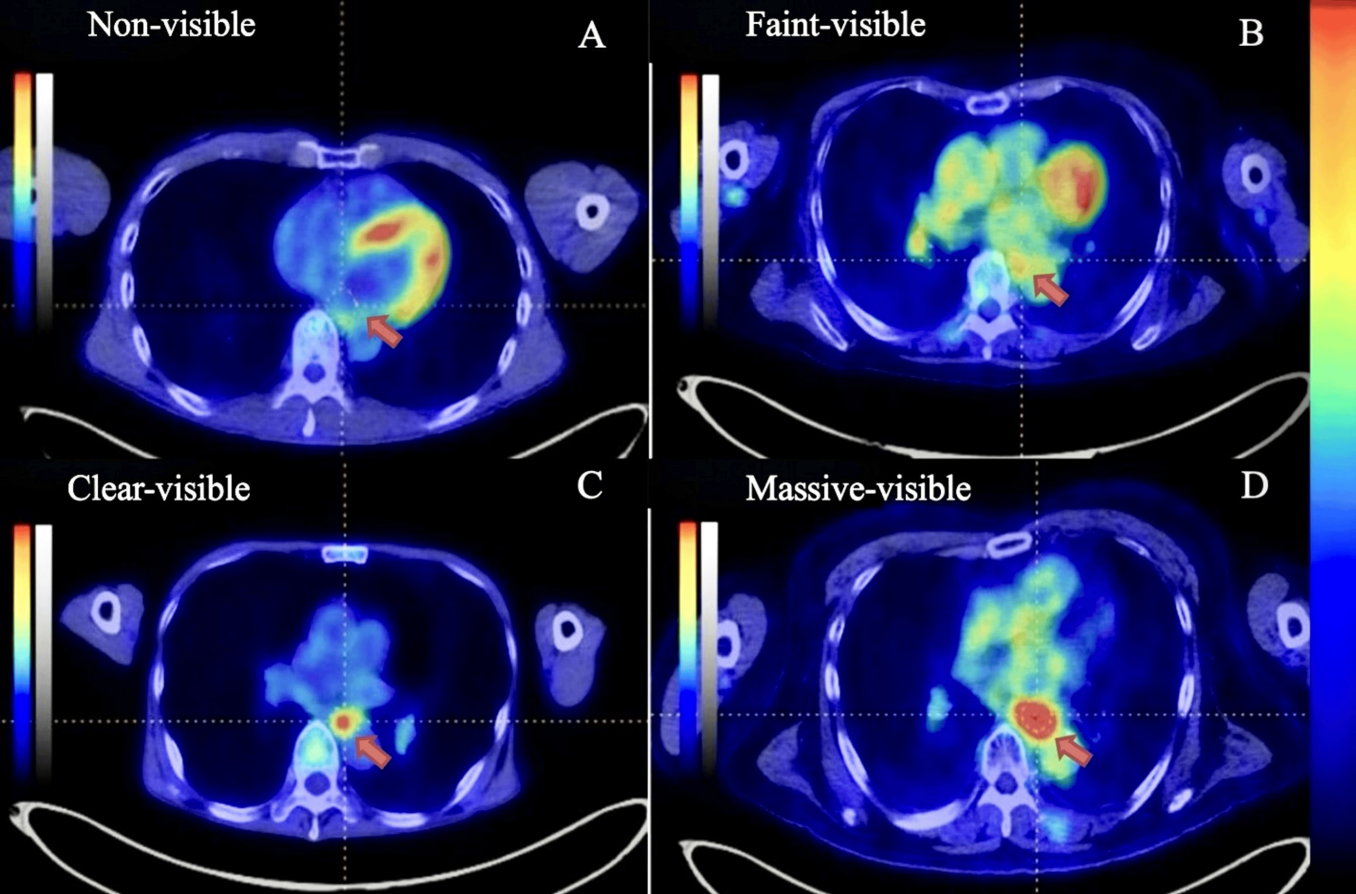
FDG visual classification of four categories Sample images are presented as a PET/CT fused image. Red color indicates high uptake of FDG (color coding is presented on the right side of the image panel. The top color indicates high, and the bottom color indicates low uptake). The panel (A) "Non-visible"; the panel (B) "Faint-visible"; the panel (C) "Clear-visible"; the panel (D) "Massive-visible". Tumors are indicated with orange arrows (but for the "Non-visible" case tumor is not visible). FDG: 18F-fluorodeoxyglucose; PET/CT: positron emission tomography/computed tomography

PET/CT images were retrieved from the hospital’s electronic archive and reviewed independently by two experienced nuclear medicine physicians (TK and AN). Lesions were classified visually into four categories: non-visible, faintly visible, clearly visible, and massively visible (Figure 1). For statistical analysis, these were consolidated into two groups: FDG-negative (non-visible and faintly visible) and FDG-positive (clearly visible and massively visible). Quantitative analyses included measurement of SUVmax, MTV, TLG, and peak standardized uptake value (SUVpeak). The mean liver SUV (SUVmean) was also recorded for reference. A threshold SUV of 3.0, consistent with previous studies, was applied to define FDG positivity. Tumors with SUVmax >3.0 were categorized as FDG-positive, while those with SUVmax ≤3.0 were classified as FDG-negative [6]. Quantitative measurements were performed using Metavol software (Hokkaido University, Sapporo, Japan).

Clinical and histopathological data

Clinical histories, treatment modalities, and follow-up information were obtained from patient records. Survival was confirmed by hospital visits or documented date of death. Data on tumor multifocality were extracted from endoscopic records, while histological differentiation (poorly, moderately, or well differentiated) was determined from surgical or biopsy specimens when available.

Statistical analysis

All statistical analyses were conducted using JMP Pro version 16.0 (SAS Institute, Cary, NC, USA). Survival was analyzed using the Kaplan-Meier method, with differences between groups compared using the log-rank test. Pearson’s chi-square test was used to evaluate associations between FDG uptake (visual or quantitative) and clinicopathological variables. Patients were followed until June 30, 2021. The primary endpoint was all-cause mortality. Patients without a recorded death were censored at their last documented hospital visit. Continuous variables were expressed as mean ± standard deviation (SD). A p-value of <0.05 was considered statistically significant.

## Results

Patient characteristics

The characteristics of the study population are summarized in Table 1. A total of 74 patients with histologically confirmed SESCC were included in the analysis. The cohort comprised 57 males (77%) and 17 females (23%), with a mean age of 68 years (range: 41-83 years). Tumor location was most frequently observed in the middle (n = 45, 61%) and lower (n = 23, 31%) segments of the esophagus, whereas upper (n = 4, 5%) and cervical (n = 2, 3%) tumors were relatively uncommon.

**Table 1:** Patient characteristics

Variable	Category	n (%)
Total sample size, n=74		
Age (years)	Median (range)	68 (41-83)
Gender (n=74)	Female	17 (23.0)
	Male	57 (77.0)
Tumor location (n=74)	Cervical	2 (2.7)
	Upper	4 (5.4)
	Middle	45 (60.8)
	Lower	23 (31.1)
Clinical T stage, cT (n=44)	cT1a	3 (6.8)
	cT1b	39 (88.6)
	cT2	2 (4.5)
Clinical N stage, cN (n=44)	cN0	38 (86.4)
	cN1	4 (9.1)
	cN2	2 (4.5)
Pathological T stage, pT (n=53)	pT1a	30 (56.6)
	pT1b	22 (41.5)
	pT2	1 (1.9)
Pathological N stage, pN (n=48)	pN0	43 (89.6)
	pN1	4 (8.3)
	pN2	1 (2.1)
Histology (n=52)	Poorly differentiated	6 (11.5)
	Moderately differentiated	32 (61.5)
	Well differentiated	5 (9.6)
	Not available	9 (17.3)
Treatment received (n=74)	Chemoradiation	3 (4.1)
	Surgery	25 (33.8)
	Endoscopic submucosal dissection (ESD)	28 (37.8)
	Radiation	4 (5.4)
	Chemotherapy	12 (16.2)
	Unknown	2 (2.7)

Treatment strategies reflected clinical stage and institutional indications. Endoscopic submucosal dissection (ESD) was performed in 28 patients (38%), surgical resection in 25 patients (34%), chemotherapy in 12 patients (16%), radiotherapy in four patients (5%), and combined chemoradiotherapy in three patients (4%). Two patients (3%) underwent other or unspecified therapeutic interventions. Detailed data on clinical stage, pathological stage, and histological differentiation are presented in Table 1.

Characteristics of the quantitative and visual groups

Quantitative evaluation of FDG PET/CT parameters demonstrated a mean SUVmax of 3.96 ± 5.51, a mean MTV of 1.79 ± 4.90, and a mean TLG of 6.42 ± 29.58. In the FDG-negative group, MTV and TLG values were uniformly zero, consistent with the absence of voxels exceeding the predefined SUV threshold of 3.0. In cases visually categorized as FDG-negative but showing minimal uptake, SUVmax was determined by manual placement of regions of interest (ROI).

Group allocation differed according to the method of assessment. Visual analysis categorized 18 patients (24%) as FDG-positive and 56 patients (76%) as FDG-negative. In contrast, quantitative classification using an SUVmax cutoff of 3.0 identified 42 patients (57%) as FDG-positive and 32 patients (43%) as FDG-negative. The distribution of cases across the four visual grading categories (non-visible, faint, clear, and massive uptake) is detailed in Table 2.

**Table 2:** Comparing visual and quantitative parameters FDG: 18F-fluorodeoxyglucose

	Massive	Clearly Visible	Faintly Visible	None	Total FDG-Positive	Total FDG-Negative
Visual analysis	2	16	23	33	18	56
Quantitative analysis	2	40	25	7	42	32

Survival analysis based on FDG PET/CT classification

**Figure 2:**
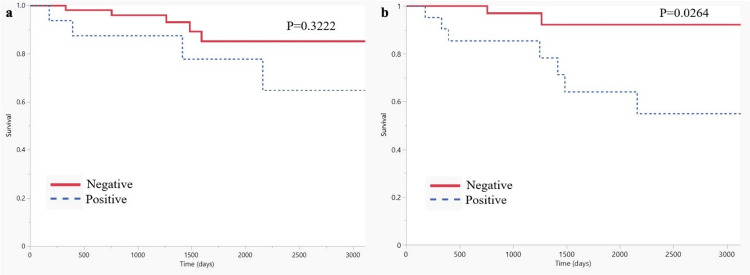
Survival analysis using visual and quantitative FDG PET classification (a) The panel shows the survival analysis using the visual grouping. (b) The panel shows the same analysis using the quantitative grouping. There is a significant difference in survival between the FDG-positive and -negative groups only when patients are grouped using the quantitative parameter. FDG: 18F-fluorodeoxyglucose; PET: positron emission tomography

**Figure 3:**
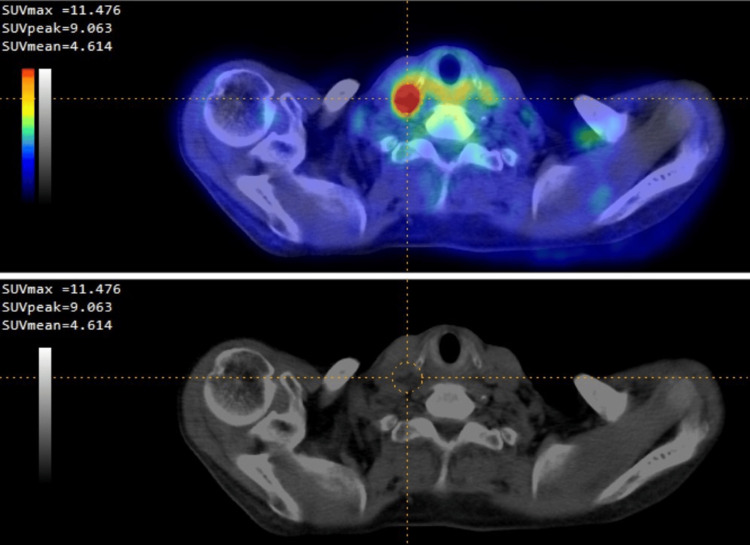
Quantitative analysis of FDG PET imaging in a patient with superficial esophageal squamous cell carcinoma using Metavol software The upper panel displays a Maximum Intensity Projection (MIP) image of FDG uptake. A red cross mark highlights areas of high FDG activity associated with the tumor. The lower panel shows the corresponding CT image with a red cross mark indicating the tumor's location. FDG: 18F-fluorodeoxyglucose; PET: positron emission tomography

The primary endpoint was overall survival, defined as death from any cause, with time zero designated as the date of the FDG PET/CT scan. Follow-up was censored on June 30, 2021, using the date of death or the last recorded clinical contact (inpatient or outpatient) for each patient. Patients were stratified into FDG-positive and FDG-negative groups according to both visual assessment and quantitative classification. Kaplan-Meier analysis demonstrated no significant difference in overall survival between FDG-negative and FDG-positive groups when stratification was based on visual interpretation (p = 0.3222, log-rank test; Figure 2a). In contrast, quantitative grouping using an SUVmax cutoff of 3.0 revealed a significant survival advantage in the FDG-negative cohort compared with the FDG-positive cohort (p = 0.0264, log-rank test; Figure 2b). This indicates that quantitative analysis provided superior prognostic discrimination compared with visual categorization. Taken together, these findings underscore the prognostic utility of quantitative PET-derived parameters in SESCC. In particular, SUVmax-based classification offers an objective and reproducible method for risk stratification that outperforms visual interpretation (Figure 3).

Relationship between FDG uptake and clinicopathological parameters

The association between FDG uptake on FDG PET/CT and clinicopathological characteristics of SESCC is summarized in Table 3.

**Table 3:** Pathological parameters and multifocality of esophageal cancer

3a. Histological differentiation vs. positivity					
Status	Poor	Moderate	Well	Total	p-value
Positive	4	20	2	26	0.5981
Negative	2	12	3	17	
Total	6	32	5	43	
3b. Pathological T stage vs. positivity					
Status	1a	1b	2	Total	p-value
Positive	16	13	0	29	0.4962
Negative	14	9	1	24	
Total	30	22	1	53	
3c. Pathological N stage vs. positivity					
Status	n0	n1	n2	Total	p-value
Positive	21	2	1	24	0.5995
Negative	22	2	0	24	
Total	43	4	1	48	
3d. Multifocal esophageal cancer vs. positivity					
Multifocality	Positive	Negative	-	Total	p-value
Yes	8	7	-	15	0.7644
No	34	25	-	59	
Total	42	32	-	74	

No significant correlations were observed between FDG findings and histological differentiation. Stratification by differentiation status (poorly, moderately, or well differentiated) yielded p-values of 0.1881 (visual analysis) and 0.5981 (quantitative analysis). Similarly, no significant associations were identified between FDG uptake and pathological TNM classification. For the T stage, p-values were 0.6253 (visual) and 0.4962 (quantitative), and for the N stage, p-values were 0.2146 (visual) and 0.5995 (quantitative). No cases of distant metastasis were detected in this cohort.

Evaluation of tumor multifocality revealed no significant association with FDG uptake (p = 0.4298 for visual, p = 0.7644 for quantitative analysis). Patient demographics also showed no significant effect: age distribution did not differ between FDG-positive and FDG-negative groups (p = 0.687 for visual, p = 0.4040 for quantitative), and sex distribution was likewise comparable (p = 0.1551 and p = 0.3595, respectively).

Interobserver agreement between the two independent readers (TK and AN) was high, with concordance of 90.5% across the original four visibility categories. Seven discrepancies were identified; after reclassification into binary groups (positive vs. negative), agreement increased to 97.3%, with only two cases of disagreement. These discrepancies were resolved through consensus. Details of interobserver variance across visual classification categories are summarized in Table 4. The observed Pearson chi-square test yielded p < 0.0001, indicating strong concordance between observers.

**Table 4:** Interobserver variance

Assessment	None	Faint	Clear	Massive	Total
None	31 (41.8%)	4 (5.4%)	0 (0.0%)	0 (0.0%)	35 (47.3%)
Faint	0 (0.0%)	19 (25.6%)	0 (0.0%)	0 (0.0%)	19 (25.6%)
Clear	2 (2.7%)	0 (0.0%)	16 (21.6%)	1 (1.35%)	19 (25.6%)
Massive	0 (0.0%)	0 (0.0%)	0 (0.0%)	1 (1.35%)	1 (1.35%)
Total	33 (44.5%)	23 (31%)	16 (21.6%)	2 (2.7%)	74 (100.0%)

Overall, FDG uptake measured by FDG PET/CT showed no significant correlation with histological subtype, pathological stage, multifocality, or patient demographics. This reinforces the finding that the prognostic utility of FDG PET/CT in this cohort is primarily derived from quantitative parameters rather than associations with baseline clinicopathological factors.

## Discussion

This study addresses an important gap in understanding the prognostic utility of FDG PET/CT in SESCC. Our findings demonstrate that patients classified as FDG-positive by quantitative SUVmax analysis exhibited significantly poorer overall survival compared with FDG-negative patients, whereas visual assessment failed to discriminate prognosis. These results underscore the superiority of quantitative over qualitative interpretation, highlighting SUVmax as an objective and reproducible parameter for risk stratification in SESCC.

Previous investigations have established FDG PET/CT as a valuable tool for staging and prognostication in advanced esophageal cancer, particularly in detecting distant metastases [7], refining stage classification, and influencing treatment allocation [8]. FDG PET has also been shown to guide surgical planning and reduce treatment-related morbidity [9], and smaller studies have suggested its potential in radiotherapy planning [10]. Collectively, this evidence confirms the central role of FDG PET/CT in the management of esophageal cancer [11]. However, its utility in superficial lesions has remained uncertain due to limitations of spatial resolution and partial-volume effects, which may obscure FDG uptake in small or thin lesions [4].

Recent studies have begun to explore this issue. Kita et al. reported that FDG PET could aid in estimating invasion depth in SESCC [12], while Nakajima et al. showed its value in refining indications for endoscopic resection [13]. Similarly, Toriyama et al. demonstrated improved accuracy in depth assessment when PET was combined with magnifying endoscopy and narrow-band imaging [14]. Notably, these studies relied primarily on visual interpretation and were unable to demonstrate prognostic significance. By contrast, our results confirm that quantitative PET-derived parameters, specifically SUVmax, carry prognostic value in SESCC, suggesting that reliance on visual assessment alone is insufficient.

Our findings are consistent with prior studies in advanced esophageal cancer, where MTV and TLG have been reported as independent predictors of overall survival [15-17]. Yuan et al. further indicated that TLG outperformed SUVmax in prognostication for locally advanced disease [17]. In SESCC, however, accurate measurement of MTV and TLG is technically challenging due to the small lesion volume and limited spatial resolution of PET. Accordingly, we adopted a threshold-based SUVmax approach (cutoff 3.0), which provided reliable stratification of prognosis in our cohort. Conceptually, FDG-positive cases in our quantitative analysis can be considered as tumors with measurable MTV/TLG, whereas FDG-negative cases correspond to non-measurable lesions. This tailored approach underscores the importance of selecting parameters appropriate to disease stage and imaging limitations.

An additional consideration is interobserver variability. Although interobserver agreement for visual assessment was generally high, discordance remained in a subset of cases, emphasizing the subjectivity of qualitative interpretation. Quantitative analysis mitigates this limitation, offering a standardized metric that improves reproducibility and reliability across readers. This advantage may also facilitate integration with artificial intelligence models, which can further refine image-based prognostication and uncover latent imaging features not apparent to human interpreters.

The present study has several limitations. The retrospective design and modest sample size restrict generalizability, and the follow-up period, although extending beyond 3000 days in some cases, remains limited for definitive survival modeling. All patients were treated at a single institution, which may introduce selection bias, and although all examinations were performed on the same PET/CT system with routine quality-control procedures, subtle scanner-related SUV variability over the long study period cannot be entirely excluded. Our findings are also limited by the absence of multivariable survival analysis, which may have further clarified the independent prognostic contribution of SUVmax and other clinical variables. Multivariate Cox regression analysis was not performed because the number of deaths was relatively small, and including multiple covariates would have risked model overfitting and unstable estimates. Furthermore, accurate quantification of MTV and TLG in very small superficial lesions remains technically challenging, which may partly explain why SUVmax-based classification provided the most robust prognostic stratification in this cohort. Prospective, multicenter studies with larger cohorts and standardized imaging protocols are warranted to validate these findings.

Despite these limitations, our results provide novel evidence supporting the prognostic value of quantitative FDG PET/CT in SESCC. Incorporating SUVmax-based analysis into clinical practice may improve risk stratification, guide individualized treatment decisions, and ultimately contribute to better patient outcomes. Moreover, the integration of quantitative PET metrics with endoscopic findings and emerging AI applications holds promise for further enhancing prognostic precision in early-stage esophageal cancer.

## Conclusions

Quantitative parameters derived from FDG PET/CT, particularly the SUVmax, demonstrate significant prognostic utility in SESCC. Classification into FDG-positive and FDG-negative groups based on SUVmax provides objective risk stratification and yields clinically relevant prognostic information. These findings support the integration of quantitative FDG PET/CT analysis into routine assessment of SESCC to guide individualized treatment strategies and optimize patient management.

## References

[1] Kim JA, Shah PM (2017). Screening and prevention strategies and endoscopic management of early esophageal cancer. Chin Clin Oncol.

[2] Smyth EC, Lagergren J, Fitzgerald RC, Lordick F, Shah MA, Lagergren P, Cunningham D (2017). Oesophageal cancer. Nat Rev Dis Primers.

[3] Kato H (1995). Diagnosis and treatment of esophageal neoplasms. Jpn J Cancer Res.

[4] Little SG, Rice TW, Bybel B (2007). Is FDG-PET indicated for superficial esophageal cancer?. Eur J Cardiothorac Surg.

[5] Brierley JD, Gospodarowicz MK, Wittekind C (2016). TNM Classification of Malignant Tumours, 8th Edition. https://www.wiley.com/en-us/TNM%2BClassification%2Bof%2BMalignant%2BTumours%2C%2B8th%2BEdition-p-x000860418.

[6] Kim SJ, Hyun SH, Moon SH (2020). Total FDG lesion number on PET/CT predicts survival of esophageal carcinoma patients with recurrence following curative surgery. Q J Nucl Med Mol Imaging.

[7] Heeren PA, Jager PL, Bongaerts F, van Dullemen H, Sluiter W, Plukker JT (2004). Detection of distant metastases in esophageal cancer with (18)F-FDG PET. J Nucl Med.

[8] Barber TW, Duong CP, Leong T, Bressel M, Drummond EG, Hicks RJ (2012). 18F-FDG PET/CT has a high impact on patient management and provides powerful prognostic stratification in the primary staging of esophageal cancer: a prospective study with mature survival data. J Nucl Med.

[9] Yoshimura S, Takahashi M, Aikou S (2020). One-by-one comparison of lymph nodes between 18F-FDG uptake and pathological diagnosis in esophageal cancer. Clin Nucl Med.

[10] Ogino I, Watanabe S, Hirasawa K, Misumi T, Hata M, Kunisaki C (2018). The importance of concurrent chemotherapy for T1 esophageal cancer: role of FDG-PET/CT for local control. In Vivo.

[11] Calais J, Dubray B, Nkhali L (2015). High FDG uptake areas on pre-radiotherapy PET/CT identify preferential sites of local relapse after chemoradiotherapy for locally advanced oesophageal cancer. Eur J Nucl Med Mol Imaging.

[12] Kita Y, Okumura H, Uchikado Y (2013). Clinical significance of 18F-fluorodeoxyglucose positron emission tomography in superficial esophageal squamous cell carcinoma. Ann Surg Oncol.

[13] Nakajima M, Muroi H, Yokoyama H, Kikuchi M, Yamaguchi S, Sasaki K, Kato H (2018). (18)F-fluorodeoxyglucose positron emission tomography can be used to determine the indication for endoscopic resection of superficial esophageal cancer. Cancer Med.

[14] Toriyama K, Tajika M, Tanaka T (2019). Clinical relevance of fluorodeoxyglucose positron emission tomography/computed tomography and magnifying endoscopy with narrow band imaging in decision-making regarding the treatment strategy for esophageal squamous cell carcinoma. World J Gastroenterol.

[15] Li Y, Zschaeck S, Lin Q, Chen S, Chen L, Wu H (2019). Metabolic parameters of sequential 18F-FDG PET/CT predict overall survival of esophageal cancer patients treated with (chemo-)radiation. Radiat Oncol.

[16] Gopal A, Xi Y, Subramaniam RM, Pinho DF (2021). Intratumoral metabolic heterogeneity and other quantitative 18F-FDG PET/CT parameters for prognosis prediction in esophageal cancer. Radiol Imaging Cancer.

[17] Yuan H, Tong DK, Vardhanabhuti V, Law SY, Chiu KW, Khong PL (2016). PET/CT in the evaluation of treatment response to neoadjuvant chemoradiotherapy and prognostication in patients with locally advanced esophageal squamous cell carcinoma. Nucl Med Commun.

